# Food Reformulation, Responsive Regulation, and “Regulatory Scaffolding”: Strengthening Performance of Salt Reduction Programs in Australia and the United Kingdom

**DOI:** 10.3390/nu7075221

**Published:** 2015-06-30

**Authors:** Roger Magnusson, Belinda Reeve

**Affiliations:** Sydney Law School, University of Sydney, Camperdown, NSW 2006, Australia; E-Mail: Roger.magnusson@sydney.edu.au

**Keywords:** salt reduction, legislation, regulation, United Kingdom, Australia, food policy, non-communicable disease

## Abstract

Strategies to reduce excess salt consumption play an important role in preventing cardiovascular disease, which is the largest contributor to global mortality from non-communicable diseases. In many countries, voluntary food reformulation programs seek to reduce salt levels across selected product categories, guided by aspirational targets to be achieved progressively over time. This paper evaluates the industry-led salt reduction programs that operate in the United Kingdom and Australia. Drawing on theoretical concepts from the field of regulatory studies, we propose a step-wise or “responsive” approach that introduces regulatory “scaffolds” to progressively increase levels of government oversight and control in response to industry inaction or under-performance. Our model makes full use of the food industry’s willingness to reduce salt levels in products to meet reformulation targets, but recognizes that governments remain accountable for addressing major diet-related health risks. Creative regulatory strategies can assist governments to fulfill their public health obligations, including in circumstances where there are political barriers to direct, statutory regulation of the food industry.

## 1. Introduction

Strategies to reduce excess salt consumption play an important role in preventing cardiovascular disease, which is the largest contributor to mortality for non-communicable diseases (NCDs) globally [1]. Some recent studies find that both low and high sodium intake is associated with increased mortality [2], calling into question the health benefits of sodium reduction [3]. However, a more well-established body of evidence demonstrates a direct, progressive relationship between sodium intake and blood pressure [4]. High blood pressure progressively increases the risk of cardiovascular disease and a number of other conditions [5], while reducing the consumption of salt reduces blood pressure and fatalities from stroke and coronary heart disease [6]. The Global Burden of Disease Study estimated that more than nine million deaths each year are caused by high blood pressure, while around 3.1 million deaths are attributable to excess salt consumption [7]. Reducing global average salt intake from current high levels (9–12 g per person per day) towards the World Health Organisation’s recommended upper daily limit of 5 g per person could therefore make a significant contribution to reductions in global mortality.

Many developed countries have introduced programs that seek to reduce population-level salt intake, including the UK, Finland, Japan, the US and Canada [8,9]. Typically these initiatives are based on a program of voluntary food reformulation by the food industry, guided by aspirational targets to be achieved progressively over time. Food reformulation is usually combined with community awareness campaigns about the risks of high salt consumption, and labeling initiatives to assist consumers to identify healthier options [9]. This paper evaluates two salt reduction initiatives: Australia’s Food and Health Dialogue—a non-regulatory partnership between the Australian government, the food industry, and public health organisations [10]—and the salt reduction program initiated by the UK Food Standards Authority in 2003. The UK initiative now forms part of the “Public Health Responsibility Deal,” a public-private partnership that aims to reduce modifiable risk factors for NCDs in the UK population [11].

Voluntary product reformulation lies at the core of both programs. However, the UK initiative is more comprehensive than its Australian counterpart, incorporating targets for reductions in population salt intake, monitoring, interpretative front-of-pack labeling, and consumer education. Both programs have had some success in reducing the sodium content of some product categories [12,13]. The UK initiative has also achieved reductions in population salt intake [14]. Nevertheless, there is significant scope in both countries to strengthen these programs, in order to further reduce population salt intake and premature death and disability from cardiovascular disease (CVD). In this paper, we advocate a stepwise approach that builds on the achievements of both programs, using the threat of “regulatory scaffolds” and increasing levels of government oversight over industry reformulation programs as an incentive for industry to accelerate their efforts to meet government targets [15,16,17].

After briefly describing the burden of disease from excess salt consumption, we assess the design and performance of the salt reduction programs in Australia and the UK. Drawing on concepts from the field of regulatory studies, we then outline a model for progressively strengthening voluntary food reformulation initiatives through the selective use of regulatory and legislative scaffolds. In the final section of the paper we apply this model to the Australian and UK salt reduction programs, setting out a series of recommendations for accelerating reductions in population salt intake in each country.

## 2. Salt Consumption, High Blood Pressure, and Cardiovascular Disease

High blood pressure affects around 32% of Australians [18], and has been estimated to be responsible for nearly 8% of Australia’s overall burden of disease, including 42% of the burden of cardiovascular disease (CVD) [19]. Apart from being Australia’s most expensive disease, CVD is the leading cause of death in Australia (responsible for 34% of all deaths) [20], and the second-largest cause of the disease burden [19]. Salt consumption in Australia averages 7–10 g/day per person [21], significantly exceeding the recommended maximum intake of approximately 6 g/day [22]. On one estimate, removing 15%–25% of sodium from processed foods could prevent 5800–9700 heart attacks and 4900–8200 strokes in Australia over a ten-year period, preventing 2000–3400 deaths [23].

Approximately one-third of UK adults have high blood pressure [24]. Cardiovascular disease affects over 13% of men and women [25], and remains the most common cause of preventable death, accounting for 29% of deaths [25]. Between 2001 and 2011, salt intake fell from 9.5 g per day to around 8.1 g/day [13]. These dietary changes were a likely contributor to significant reductions in stroke (42%) and ischaemic heart disease (40%) during this period [26]. Despite this, in 2011, over 70% of the population exceeded the UK recommended maximum salt intake of 6 g/day. In men and women aged 19 to 64, average salt intake was 8.1 g/day and 6.8 g/day, respectively [14]. For those aged over 65, average salt intake was even higher for men (8.3 g/day), although lower for women (6.4 g/day) [27].

In most developed countries, processed foods and ready-made meals are estimated to make the largest contribution to salt intake, comprising around 75%–80% of individual consumption [28]. Products that do not taste particularly salty can have unexpectedly high levels of sodium, such as muffins or bread [29]. The hidden salt content of processed products makes it difficult for consumers to monitor their salt intake and to maintain long-term dietary changes, particularly when combined with the confusing array of nutrition labeling on processed foods [30]. Individuals also develop high-salt taste preferences through exposure to high-salt foods, making it more difficult to adjust to reduced-sodium products [31]. Nevertheless, significant variations observed in the salt levels of comparable products, such as cheese and processed meat, suggests that step-wise reductions in the salt content of processed foods is a feasible approach to reducing sodium intake [32,33,34].

## 3. Evaluating Australia’s Food and Health Dialogue

The Australian government established the Food and Health Dialogue in 2009 as a public-private partnership [35], with government agencies, the food industry and public health organisations represented in its governance structure [36]. The Dialogue aims to improve the nutritional quality of processed foods through product reformulation [35], and includes 20 targets for salt reduction across nine food categories, with a timeframe for each category. These include targets for bread, breakfast cereals, simmer sauces, processed meat, soups, savory pies, potato/corn/extruded snacks, cheese, and savory crackers [37].

Some targets take the form of a percentage reduction in sodium levels in products that exceed a nominated threshold amount; for example, a “10% reduction in sodium across wet savoury pies with sodium levels exceeding 400 mg/100 g” [37]. Others are expressed as a simple maximum (for example, 710 mg/100 g for cheddar and cheddar style variety cheeses), or consist of an average salt reduction target for a specific food category, combined with an upper limit [37]. Participating companies decide which products to reformulate and the amount of sodium reductions that is needed each year in order to meet the target within the agreed timeline. The Dialogue establishes action plans for each product category, and participants are expected to report annually against the commitments that they make under these plans [38].

Independent research shows that the Dialogue has made some progress in reducing the sodium content of targeted food products. One study of sodium levels in breads, breakfast cereals and processed meats in Australian supermarkets between 2010 and 2013 found an average sodium reduction of 9% in bread, 25% in cereals, and 8% in processed meats [12]. Although the proportion of products meeting the 2013 targets rose during the study period, sodium content varied widely within each product category. There were also substantial differences in the extent to which participants met the Dialogue’s targets. For example, compliance with the target for processed meats ranged from 14% to 90% [12]. Other research suggests that the Dialogue has not produced consistent improvements in the nutritional quality of targeted products, and none of the Dialogue’s targets have been achieved completely [36].

One explanation for the Dialogue’s limited impact may be its incomplete coverage of the food industry, as well as significant variations in participants’ reformulation efforts [12]. Approximately 42 companies participate in the Dialogue, including major supermarket chains Aldi, Coles, and Woolworths, and transnational food manufacturers such as Unilever and Nestlé [38]. While those companies that have joined the scheme represent the majority of market share for each targeted product category, many companies have not joined the Dialogue, and many products remain unaffected by any targets. For example, Dialogue participants account for more than 80% of the market share for breads [39], but only 60% of the market share for cereals [40]. Thus, the Dialogue covers a significant proportion of cereal manufacturers, but 40% of cereal products still remain outside the program. While the Dialogue established a quick serve restaurant (*i.e.*, fast food) engagement strategy in August 2012 [41], it has not set any targets for the food service sector, and there is no evidence of effective engagement with fast food companies.

**Figure 1:**
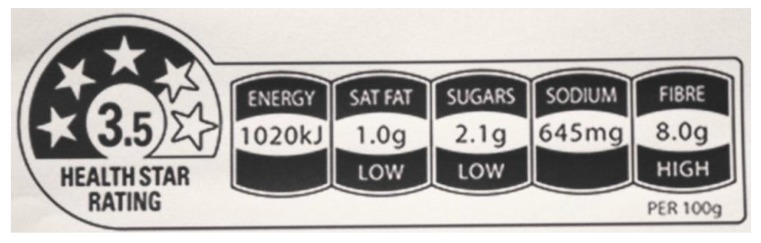
The voluntary, front-of-pack “Health Star Rating” label (Australia) [43] (Health Star Rating trademarks are owned by the Commonwealth of Australia.

The design of the Dialogue is weak when compared to other, more successful salt reduction initiatives. In contrast to the UK approach, there is no overall target for salt reduction at the population level, nor are food reformulation efforts supported by a consumer education campaign [12]. In December 2014 the Federal government launched the “Health Star Rating” labeling system, which was initiated by the previous Federal Labour government in collaboration with State and Territory governments, the food industry, and public health and consumer organisations. Health star rating labels aim to assist consumers to choose healthier products based on an overall assessment of the relative levels of saturated fats, sugars and sodium in processed food products (Figure 1) [42,43]. Manufacturers can indicate that a product is low in one or more of these nutrients [44], where the product meets requirements set out in the Australia New Zealand Food Standards Code [45]. However, implementation of the system is voluntary, with a rollout period of five years, and the food industry has successfully lobbied to retain the use of its Daily Intake Guide label, which is designed to avoid adverse judgments about levels of nutrients (including salt) in food [42,46].

The Dialogue has adopted a much smaller number of reformulation targets in comparison to the UK initiative (20 targets in nine food categories *vs.* 85 targets in 30 product categories), and fails to cover some key categories of processed food, such as ready meals [47]. The targets themselves are often weaker than those found in other programs [48]. For example the 2012 UK target for the salt content of simmer sauces was a mean sodium content of 330 mg/100 g [49]. By contrast, the target set by the Dialogue was far less onerous: a 15% reduction in the salt content of sauces containing more than 420 mg/100 g of sodium, between 2011 and 2014 [50].

The Dialogue’s governance processes contain significant limitations. The Department of Health does not monitor changes in the salt content of foods in targeted categories, and there is no reporting of the impact of reformulation on consumers’ purchasing patterns [36]. The Food Standards Authority Australia New Zealand (FSANZ) has been engaged to assess the impact of the Dialogue on population salt intake, but it has yet to release any findings [38]. The Dialogue requires self-reporting by participants, but it does not publish detailed information on companies’ progress in meeting salt reduction targets, undermining the transparency and accountability of the scheme [36]. In summary, there is “no clear reporting of outcomes, no systematic baseline data collection and little quantitative reporting of progress…” [36]. Judged against the goal of reducing the preventable burden of cardiovascular disease within a medium-term timeframe, the Dialogue’s salt reduction program is failing [36].

## 4. Evaluating Salt Reduction Initiatives in the UK

In 2003, the Food Standards Authority (FSA) set the objective of reducing population salt intake from 9.5 g per person to 6 g per person by 2010—a reduction of 40% [51]. In 2006, following consultation with the food industry, the FSA published salt reduction targets for 85 food types in 30 different product categories [52]. The FSA also developed an interpretive food-labeling scheme that displayed nutrient levels for fat, saturated fat, sugar and salt in a “traffic light” format on the front of food packages [51].

The FSA’s food reformulation strategy was supported by a four-phase education campaign that aimed to improve consumers’ knowledge of the links between salt and health, to increase their demand for low-salt products, and to educate them on how to reduce their salt intake [51]. The FSA monitored the impact of the initiative using repeated, national 24-h urine surveys [53,54], and established a processed Food Databank that enabled it to track the salt levels in food products over time [54].

Between 2004 and 2011, the UK program achieved significant reductions in salt levels in key products including breakfast cereals (57%), and sweet biscuits (25%) [13]. Following the FSA’s consumer education campaign, 43% of adults reported making an effort to cut down their salt intake, compared to 34% before the campaign started. Repeated household surveys carried out between 1997 and 2007 also show a steady decline in salt added at the table [55]. Overall, between 2001 and 2011, salt intake in the UK fell from 11 g/day to 9.7 g/day in men, and from 8.1 g/day to 7.7 g/day in women [14].

Despite these reductions, the UK initiative was not on track to meet the 6 g per person target for daily salt intake set in 2006. In 2009, the FSA published a revised set of targets to be achieved by 2012 [51]. However, in 2010 the newly elected coalition government transferred nutrition policy from the FSA to the Department of Health, and rolled the salt reduction program into the Public Health Responsibility Deal—a public-private partnership for health between government, businesses, NGOs, and public health organisations [51,56].

The Deal is based on five networks—food, alcohol, workplace health, physical activity, and a behavior-change group that supports the other four networks [57]. Participants are required to sign up to a set of general “core commitments” and supporting principles that underpin the Deal generally, and in addition, to sign onto one or more collective pledges that set out the specific actions that participants agree to take within one or other of the four action areas [58]. Participants are expected to write “delivery plans” that describe the activities undertaken in support of the collective pledges they have signed, to monitor their progress against agreed indicators, and to report annually on progress [58,59]. The Department of Health publishes annual updates on the Deal’s website [59], and monitors progress in salt reduction through the National Diet and Nutrition Survey, urinary sodium surveys, and available market data [60].

The Salt Reduction Pledge commits signatories to meet the FSA’s 2012 salt reduction targets, which are expressed either as sales-weighted and process averages, or as a maximum salt level for all products within a particular category [61]. In July 2012 the Deal launched three new pledges focusing on the catering sector [62], covering chef training and kitchen practices, reformulation of key menu items, and procurement practices [63,64,65,66].

In March 2014, the food network published a new salt pledge setting maximum per-serving targets for meals purchased “out of home” [67]. The pledge covers 11 food categories and 24 sub-categories, based on the ten most popular take-away food dishes in the UK, including chips, fries, and pizzas [67]. The network also revised existing 2012 targets and set more demanding targets for 76 categories of products, to be met by 2017 [68]. Because some targets are technically difficult to achieve, companies are considered compliant when 95% of their products or volume sales meet the targets, and they have attempted to reduce the salt content of their remaining products or volume sales [68]. However, new products introduced into the market cannot exceed the current maximum target for the relevant category [68]. In the year since the 2017 salt targets were announced, around 60% of manufacturers and retailers have signed on [69], although this does not include large companies including Unilever, Kellogg’s and McDonald’s [56].

The UK’s collaborative, government-led approach has been a model for salt reduction initiatives in other countries [51]. The UK strategy includes an impressive number of salt reduction targets and has successfully engaged food retailers, caterers and manufacturers, thus reducing salt use across the entire food chain [51]. The extension of pledges and targets to the catering sector is both novel and necessary. Nevertheless, some processed food categories still have very high salt content, with significant variation within product categories. One study of 23 different take-away meals found that a single portion of an average meal contained more than half of the FSA’s 6 g/day target, with some meals providing more than 200% of recommended daily salt intake [70].

**Figure 2:**
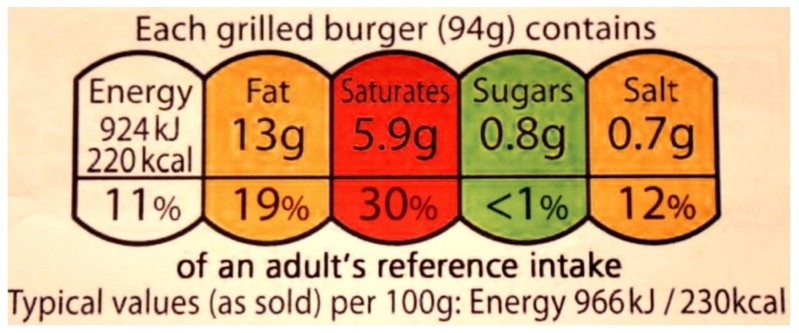
The voluntary, hybrid, front-of-pack nutrition label incorporating percentage reference intakes (daily guideline amounts) with color coding (United Kingdom).

As in Australia, the format of front-of-pack nutrition labeling has been highly controversial in the UK. In June 2013, British Health Ministers introduced a harmonized, hybrid front-of-pack system that incorporates percentage reference intakes together with color coding (Figure 2) [71]. However, in October 2014 the European Commission formally opened infringement proceedings against the UK for recommending (voluntary) use of the scheme [72]. This action followed complaints from the food industry and European Union (EU) member states that the scheme hampered the marketing of some products within the region, thus breaching EU law on the free movement of goods [72].

The number of participants to the Deal has increased steadily, with 776 partners listed on its website as of June 2015 [73]. The 2012 salt reduction pledge has 78 participants, but a number of large companies have not joined [74], and the catering-related salt reduction pledges have a much smaller number of participants (ranging from between 9 to 15) [64,65,66]. The Deal has experienced difficulty in securing comprehensive membership from all sectors of the food industry [75], and in convincing caterers to join the salt reduction pledges [76]. In the absence of sanctions for refusing to join the Deal, or to comply with commitments made under it, non-participants can “free-ride” on the benefits generated by participating companies.

Supporters of the Deal cite its high level of transparency, with commitments and pledges made available for external scrutiny [57]. Government and civil society are represented in the Deal’s networks and steering committees, but industry actors comprise the largest proportion of its governing bodies [56,75]. Panjwani and Caraher argue that the absence of disincentives for non-participation in the Deal enabled industry to significantly weaken the obligations owed under the pledges in exchange for their participation [75]. Industry influence over the calorie reduction pledge resulted in the removal of quantitative metrics for measuring progress, re-framing industry’s commitment to report on “progress” to a commitment to report on “actions” taken, and including new product development, education and health promotion (rather than product reformulation) as examples of actions taken in support of the pledge [75]. Effective self-regulation requires independent monitoring and evaluation of “clearly defined, quantifiable targets with time frames, and with a specified baseline for the purpose of comparison” [77]. However, in the absence of consequences for underperformance or non-participation, there is little pressure on businesses to stretch themselves to achieve targets voluntarily [78].

## 5. Accelerating Progress of Salt Reduction Initiatives: Insights from the Field of Regulatory Studies

Strengthening the performance of voluntary salt reduction initiatives could significantly reduce the burden of cardiovascular disease in the United Kingdom, Australia, and other countries where these programs operate. Several countries have taken a legislative approach to salt reduction, and imposed mandatory upper limits for salt either in particular products (e.g., bread), or for a wider range of food categories that contribute to excess salt intake at the population level. For example, in 2013, South Africa introduced regulations that impose maximum salt levels for 13 food categories, including bread, breakfast cereals and porridges, butter and fat spreads, processed meat, savory snacks, and potato crisps [79,80]. Modeling studies indicate that legislation to reduce salt limits in food is both effective and cost-saving [81,82], especially when combined with complementary strategies such as health promotion through the mass media [82,83]. On the other hand, in circumstances where statutory regulation is not achievable [15,16,17], this does not mean that the only alternative is industry self-regulation; rather, a variety of regulatory options exist that could be used to accelerate the progress of salt reduction programs.

The field of regulatory studies provides important insights into new forms of public health governance through its elaboration of regulatory instruments and strategies that governments can use to guide industry behavior. Regulatory scholars refer to the contemporary era as one of “regulatory capitalism”—an era characterized by regulatory complexity and fragmentation, and a division of regulatory tasks between a range of public and private actors [84,85,86,87]. The characterization of the regulatory environment as complex might seem counterintuitive, given that globalization has been a major economic force responsible for challenging the legitimacy of state regulation and supporting a “neo-liberal agenda” [88]. Neo-liberalism, in turn, is variously associated with a commitment to: reducing the regulatory burden on business; greater deference to markets; heightened emphasis on “personal responsibility”; and the dismantling of the welfare state through the shifting of social responsibilities back to the private realm.

Despite this, regulatory scholars argue that growing privatization and globalization has in fact heightened the demand for regulation [85,86], although it is shared between both the public and private sectors, and evident through a more complex series of forms that can be ordered according to the degree of government influence involved [87]. For example, Julia Black divides self-regulation into “pure” self-regulation, “coerced” self-regulation (developed in response to the threat of statutory regulation), and “sanctioned” self-regulation, where businesses formulate rules that are formally approved by government [89]. Finally, under “mandated” self-regulation (also known as co-regulation), businesses develop private rules within a framework of objectives and oversight established by the state [89].

In addition to describing the wide array of regulatory forms that governments can deploy, regulatory theorists have addressed the normative question of *when* direct, statutory regulation is justified. Ayres and Braithwaite’s theory of responsive regulation proposes an incremental approach in which governments begin by encouraging and monitoring voluntary measures, but move towards more coercive measures if industry fails to cooperate in achieving public policy objectives [90,91]. Extensions of the theory suggest that governments should tailor their regulatory approach to the characteristics of the industry concerned, the policy objectives to be achieved, and the political, social and economic context of regulation, as well as to industry’s willingness to cooperate [92]. Governments should also consider opportunities for using multiple, complementary instruments to address regulatory problems, rather than relying upon a single form of regulation operating in isolation [93,94].

The field of regulatory studies provides some helpful concepts and insights that can be adapted by governments to strengthen the performance of voluntary food reformulation initiatives. First, despite the complexity of regulatory forms in the era of regulatory capitalism, the state should remain accountable for protecting the public’s health, and for the performance of public health initiatives [95,96]. In fulfilling that responsibility, governments have considerable flexibility in the design of accountability mechanisms that meet their objectives, rather than a simple choice between introducing a new legislative regime, or defaulting to industry-initiated, self-regulatory initiatives [96]. In circumstances where government permits the private sector to respond to nationally significant health risks through self-regulation, it remains responsible for holding industry accountable for its performance, and for ensuring that regulatory processes are effective in achieving public goals [89,96,97,98,99]. The state’s role in regulation has been described as “steering” rather than “rowing”; that is, guiding the direction and evaluating the performance of self-regulatory arrangements, rather than directly prescribing and enforcing mandatory standards [100]. However, it must be willing to intervene more directly in voluntary programs where industry proves unresponsive to state regulation “at a distance”.

In democratic societies, periodic elections and other democratic processes help to ensure transparency, and also play a role in holding the state accountable for its actions to protect the public’s health (or for its failure to do so). By contrast, transparency and accountability mechanisms tend to be lacking in voluntary initiatives involving the food industry [75,101], as companies are primarily motivated to maximize shareholder returns and will pursue public health objectives only to the extent that it benefits overall financial performance, or to the extent required by legislation. The responsive regulatory approach we advocate in this paper encourages governments to make intelligent use of both motivations. In many cases, companies will want to avoid a legislated regime, which may partly explain why industry developed a voluntary code in the first place. The threat of legislation represents a powerful “pull” factor that increases incentives for higher levels of compliance with industry standards; at the same time, the introduction of carefully-chosen “regulatory scaffolds” around the code or standard—represent “push” factors towards compliance. The combination of these push and pull factors represents a new and distinctive approach to public health regulation.

Regulatory theory illustrates the wide variety of regulatory tools that are available to increase the accountability of the food sector, and to enable government to fulfill its “steering” role in public health. These include setting goals and indicators for success, improving accountability and transparency of voluntary standards, including through independent administration of industry codes, and requiring independent monitoring and evaluation of the extent to which voluntary initiatives are successful in achieving public goals. Table 1 collates these ways of strengthening under-performing voluntary schemes into a framework of regulatory or legislative “scaffolds” that provide options for governments to draw on as needed. As discussed below, this framework addresses three aspects of regulatory design: first, the regulatory content of industry codes and standards (including specific goals, terms, definitions and conditions); second, regulatory processes (e.g., administration, monitoring and evaluation); and third, enforcement (*i.e.*, the use of “carrots” to encourage compliance and “sticks” to deter poor performance) [15,17,102]. The framework illustrated in Table 1 is not limited to food reformulation, but could be used to strengthen other industry-initiated regulatory process, including the regulation of food advertising to children [103,104].

**Table 1:** A framework of “regulatory scaffolds” for strengthening the design and performance of food reformulation initiatives.

*Regulatory Component*	*Actions to Be Taken*
*Regulatory Category or Dimension*	
**Substantive content**	
The goals of industry codes	Clearly identify the goals the initiative is intended to achieve; include measurable targets for evaluating overall performance across a defined timeframe.
Terms, definitions and exceptions in industry codes	Define key terms and definitions used in initiatives; specifically identify any exceptions.
**Regulatory processes**	
Administration	Grant administration of the scheme to an independent, accountable body, e.g., a committee with equal representation from government, industry, and public health organisations, with each member’s roles and responsibilities clearly identified in writing.
Monitoring	Conduct independent, transparent and comprehensive monitoring of the scheme, using baseline data and a set of measurable, time-bound process and outcome indicators, and accompanied by public reporting of the results.
Review	Undertake regular, independent, external reviews, using baseline data and performance indicators that can be used to measure the initiative’s success in achieving its objectives; publicly report the results of any reviews.
**Enforcement**	
Incentives for compliance	Provide incentives that motivate participants to comply, e.g., positive publicity, subsidies for research and development, or a promotional labeling scheme.
Deterrents for non-compliance	Provide for a wide range of sanctions that deter non-compliance by participants and free-riding by non-participants, e.g., “naming and shaming”, fines, and expulsion from the scheme; threaten escalation to more coercive regulatory options if voluntary initiatives fail to produce significant improvements in companies’ performance.

Strong government leadership is a necessary condition for strengthening performance of voluntary or non-statutory food reformulation initiatives. Ideally, this will be reflected in over-arching goals, targets, and indicators for evaluating the performance of food companies and other participants. Government must also position voluntary schemes within a policy framework that sets out the role that businesses are expected to play in achieving public health goals and creates a credible expectation that more direct forms of regulation will be imposed if industry under-performs. Measurable targets are necessary to ensure that the overall progress of food reformulation initiatives towards the achievement of national goals can be evaluated objectively, and in order to compare the relative performance of participating companies (benchmarking) [105,106].

The second dimension of regulatory design relates to regulatory processes themselves. Independent monitoring and evaluation of the operation and performance of food reformulation initiatives are particularly important for their role in increasing transparency and accountability for voluntary commitments [103]. Monitoring also provides the evidence base that justifies escalating the level of regulatory intervention when voluntary programs fail to make timely progress towards public health objectives. The overall performance of voluntary regulatory schemes should be subject to independent, regular review, guided by baseline data and performance indicators that can be used to judge the scheme’s level of performance or success. Along with monitoring, independent review is critical to enhancing the responsiveness of private initiatives to external stakeholder concerns [102]. The government can also foster transparency by publicizing annual reports and the results of reviews on the scheme’s progress [102].

The third dimension of regulatory design highlighted in Table 1 is enforcement: the provision of “carrots” that encourage companies to join voluntary schemes and to change their products in line with their commitments, combined with “sticks” that deter non-compliance and prevent free-riding on voluntary schemes [107,108]. Governments can encourage compliance through education, collecting information, and publicity that praises well-performing companies [102,109,110], as well as tax breaks or other economic benefits for participants that achieve regulatory objectives [31]. Governments may also “name and shame” companies that refuse to participate in the scheme, expel repeat offenders, or refer serious instances of breach to government regulators [91,105]. Negative publicity may be effective with public companies that have corporate reputations to protect and rely on institutional investors. As one organisation commented, “public shaming is like being ‘dumped into custard—it’s a soft landing, but it sticks’” [102].

Studies of regulation suggest that the threat of government regulation often acts as the prompt for the creation of voluntary industry initiatives [93]. Where voluntary schemes fail to achieve satisfactory performance, governments will be justified in adopting a responsive regulatory approach and escalating towards more coercive regulatory measures [91,111]. In the final sections of this paper, we apply the concept of regulatory scaffolds to the Australian and UK salt reduction strategies, illustrating how governments in both countries could progressively increase their level of intervention in salt reduction initiatives in response to the under-performance of these initiatives in achieving feasible targets for reductions in population salt intake.

## 6. Strengthening the Food and Health Dialogue

We propose a three-phase strategy for strengthening the Dialogue, adopting a responsive regulatory approach, and using legislative scaffolds as necessary to strengthen: the content of regulatory requirements, regulatory processes, and enforcement. Phase 1 would aim to preserve a collaborative and voluntary approach to food reformulation. However, industry efforts would now take place within the context of overall targets for reductions in population salt intake for which identified sectors of the food industry would be held accountable. Under Phases 2 and 3, governments would escalate regulatory control over the activities of food businesses if significant improvements in the food supply were not achieved in Phase 1. In extreme cases, this process of escalation could culminate in the direct regulation of individual non-complying companies.

### 6.1. Phase 1

The Food and Health Dialogue lacks the basic foundations of an effective reformulation program, including a national target for population salt reduction (see Table 2). A national target provides a clear expression of the government’s commitment to reducing population salt intake, and the ultimate standard against which the performance of the Dialogue should be measured. Thus, a stronger reformulation program would begin with the creation of an overall target for population salt reduction, such as 6 g per day, accompanied by a timeframe for achieving it. Independent evaluation of the performance of the Dialogue would also require the Department of Health to collect comprehensive baseline data on population salt intake [53], and to create a food databank recording baseline average salt levels across the food categories and sub-categories for which targets would be set. This would permit the tracking of salt levels within each food category, evaluation of progress towards reduction targets, and monitoring of reductions in population salt intake.

Once a national target is established, and following consultation with food industry actors, government would allocate responsibility for achieving a specified share of the national target to those food industry sectors that are significant contributors to excess salt intake, including processed food manufacturers and food retailers—particularly supermarkets and chain restaurants. If at least three quarters of dietary salt is added during manufacture [28], then it is appropriate for the food industry to be held accountable—collectively—for achieving at least three quarters of the reductions in salt intake that are necessary to achieve the national target [15]. However, government would still need to allocate responsibility between processed food manufacturers, supermarkets, chain restaurants, and possibly other industry sectors.

**Table 2:** Key features of a step-wise, responsive regulatory approach to strengthening voluntary national salt reduction schemes.

**Pre-Requisites to an Effective Product Reformulation Program**			
Government should: - Commit to a national target for population salt reduction - Collect baseline data on population salt intake - Develop a food databank recording baseline average salt levels across food categories and sub-categories for which targets will be set - Allocate responsibility for achieving a specific share of the national target between processed food manufacturers and food retailers			
	**Substantive Content of Participants Obligations**	**Regulatory Processes**	**Participation and Enforcement**
Phase 1	Wide-ranging, aggressive reformulation targets are created, which if achieved, will enable food manufacturers and retailers to meet their share of the national target.Maximum salt caps are introduced, particularly for product categories that contribute significantly to excess salt intake, and potentially for new products introduced into the market. Compliance with reformulation targets and salt caps remains voluntary. However, participating companies are required to report on specific actions taken to meet targets and commitments.	Governance structures for the salt reduction scheme are strengthened by increasing the level of representation by government, consumer and public health groups.	Food manufacturers, retailers and caterers that contribute the greatest amount of salt to the food supply are identified and asked to join the scheme. Government and/or scheme administration engages in a targeted recruitment drive. Department of Health threatens industry with mandatory participation in Phase 2 if there is insufficient compliance/low levels of participation. Companies that fail to meet targets and commitments are “named and shamed”; high achievers are praised. Government sets out a timetable for legislative action if progress falls behind minimum-stated level of achievement within a given timeframe.
Phase 2	Average salt reduction targets and salt caps apply specifically to each manufacturer’s product portfolio (rather than collectively to all participants).Reformulation targets and caps remain voluntary, but mandatory high-salt warning labels apply to non- complying products (e.g., mandatory traffic light labeling).	Administration of the scheme is transferred to an independent government agency, which is given a statutory mandate to implement and enforce the program.	Companies that fail to prepare action plans and submit annual reports in a timely fashion are penalized (e.g., fines).
Phase 3	The independent regulator could set mandatory targets for particular product categories where participants fail to make adequate progress.Dept. of Health (or independent regulator) requires under-performing companies to enter into enforceable agreements to implement reformulation plans, with company-specific targets for product lines, interim targets, and sanctions for non-compliance.Complying companies would remain under Phase 2.		
Phase 4	Government introduces mandatory salt limits for sales-weighted averages and maximum salt caps for a wide range of processed and restaurant food categories.		

**Figure 3:**
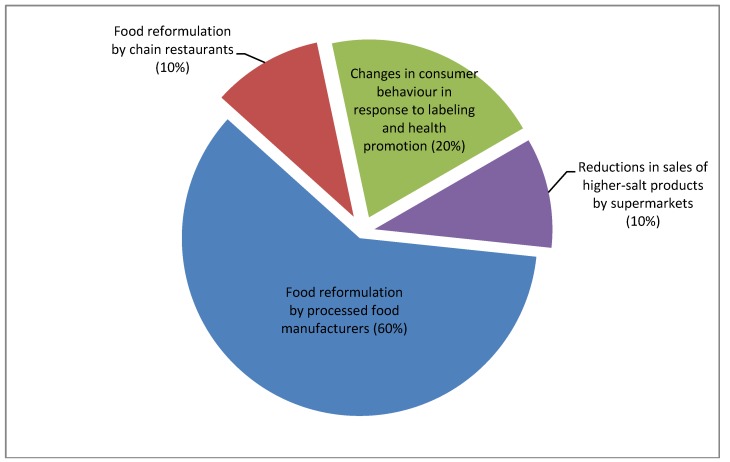
Pie-chart showing indicative allocation of responsibility for reducing population salt intake between consumers, food manufacturers, supermarket chains and chain restaurants.

Figure 3 illustrates a policy environment in which key food industry actors are held accountable for 80% of the reductions necessary to achieve the national target, with changes in consumer behavior being responsible for the remaining 20%. Health promotion and food labeling initiatives could support consumers to reduce their salt intake, including by choosing lower-salt products, and adding less salt to food at table. However, the cost of mass media campaigns may make it more cost effective for governments to focus their efforts on lower-cost social media, and on creating incentives for the food industry to take its food reformulation targets more seriously.

In Phase 1, the Department of Health would strengthen salt negotiations by reframing the Dialogue as a joint government/industry “Compact” and inviting all major food manufacturers, food retailers and restaurant chains to join. Although a relatively small number of companies account for a large proportion of food sales (with supermarket chains Coles and Woolworths dominating food retail sales) [112], smaller manufacturers and retailers would also be encouraged to sign on to the targets set out in the Compact, particularly smaller-sized businesses selling high-salt products.

The extent to which food industry accountability should be focused on supermarkets, or on food manufacturers, or be shared more widely between food manufacturers and food retailers, remains a matter of debate [113,114]. For example, supermarkets not only have complete control over their own “home” brands, but could favor lower-salt products for shelf space and use their commercial influence over their supply chains to require suppliers to reformulate their products and to meet salt reduction targets. Reformulation targets for supermarkets and other food retailers could be expressed as a percentage reduction from a baseline representing current, average salt levels passing through cash registers, across key product categories [114]. Below, we illustrate a responsive regulatory approach to food reformulation directed specifically at food manufacturers and chain restaurants.

To strengthen the accountability of the Compact to external interests, the Department of Health would convene a High-Level Steering Committee (HLSC) comprising an equal balance of food industry representatives, relevant government agencies, public health organisations, nutrition experts, and consumer groups. The HLSC could convene working parties to set targets for each food category, and would have overall responsibility for reviewing draft targets and agreeing on a timeframe for achieving them. The reformulation targets approved by the HLSC would need to be sufficiently aggressive and to cover a sufficiently wide number of food categories so that achievement of the targets would fulfill that share of the national target for which food manufacturers were accountable (60% of the national target, in the example in Figure 3).

In addition to targets for average salt reductions to be achieved collectively by food manufacturers for each food category, the HLSC would also need to agree on maximum salt caps to ensure that food companies do not game the system by introducing low-salt products in order to “off-set” high-salt products in their portfolio. Alternatively, salt caps could target high-volume product categories that account for the largest share of excess salt intake, or alternatively, new products introduced into the market [68]. In Phase 1, compliance with average salt reduction targets and salt caps would be voluntary. However, all new products introduced into the market that exceeded these caps would be required to bear a high-salt warning label: a significant disincentive to increasing salt levels in existing product categories. While mandatory, high-salt warning labels are currently novel; they may become more common in future. For example, the New York City Health Department has recently proposed a mandatory warning label on dishes sold by chain restaurants that contain more than 2300 mg sodium (5.75 g salt) [115].

In Phase 1, participants would be requested to prepare and file action plans and to report annually on their progress in bringing their products into compliance with relevant targets. The publication of both sets of documents on the Dialogue’s website would enhance transparency and enable external stakeholders to either praise or “name and shame” participants depending upon their performance. The government could also monitor industry performance and create a publicly accessible list of non-complying products. Negative publicity from this list would be a powerful incentive for companies to reformulate their products.

### 6.2. Phase 2

The failure of food manufacturers to achieve their share of the national salt reduction target could trigger the introduction of more intrusive controls. Under Phase 2, average salt reduction targets would apply specifically to each manufacturer’s product portfolio, rather than collectively to food manufacturers as a whole; compliance would also become mandatory for food manufacturers whose sales exceeded a minimum market share within each product category. For some companies with highly specialized product portfolios (e.g., those producing one form of condiment), compliance could be measured in terms of steady reductions in salt content in line with the category average. Smaller companies selling high-salt products could also be required to join the Compact on the basis of their product portfolio. Accountability could also be strengthened by transferring administration of the salt reduction compact to an independent body [116], which would also be given a statutory mandate, including the power to acquire information, and in cases of blatant non-compliance, to accept court-enforceable undertakings and to issue orders preventing the sale of products breaching salt caps.

Stronger regulatory processes under Phase 2 would be accompanied by more significant sanctions for non-compliance. Although compliance with salt caps for existing products would remain voluntary, companies would be penalized for failing to prepare action plans and annual reports in a timely fashion. Companies that failed to reformulate their products to meet salt reduction targets within the nominated period would be required to include a mandatory high-salt warning label on their products.

### 6.3. Phase 3

Phase 3 would involve targeted regulation of under-performing companies, following an independent audit of the scheme. If auditing revealed that companies were failing to implement action plans to meet average and maximum salt levels, an independent regulator would have the power to require companies to give court-enforceable undertakings, with financial penalties for non-compliance [117,118,119].

## 7. Improving the UK’s Salt Reduction Program under the Responsibility Deal

The UK’s salt reduction program, now part of the Responsibility Deal, has the basic prerequisites of a successful reformulation program (see Table 2). These include a national target for population salt reduction, baseline data on population salt intake, and baseline data for measuring reductions across relevant food categories. The UK program also has a larger number of participants, a broader range of reformulation targets, and more sophisticated monitoring and reporting mechanisms than Australia’s Food and Health Dialogue. Nevertheless, weaknesses in the structure and operation of the Deal undermine its capacity to recruit new participants to the salt pledges, to ensure that companies reformulate their products to meet the pledge targets, and to raise targets over time without losing participants.

Voluntarism and lack of accountability are the key weakness of the Deal. In particular, the absence of consequences for failing to join weakens the content of the pledges that are made, and if targets and requirements “stretch” participants too far, participants can simply refuse to cooperate. In addition, the absence of accountability for the food industry’s collective failure to meet the existing targets severs the strategic link between the Deal’s targets, and the process that is meant to achieve them.

### 7.1. Phase 1

A responsive regulatory approach enables governments to increase the incentives for food businesses to join the Deal’s salt reduction pledges and to make the achievement of salt reduction targets a genuine priority for their operations, while initially avoiding coercive obligations. In Phase 1, we propose four recommendations that would: strengthen government leadership over the Deal, strengthen the integrity of the Deal’s performance monitoring process, increase the incentives to join the salt reduction pledges, and the disincentives for non-compliance. The purpose of these “scaffolds” is to accelerate voluntary actions by food businesses to reduce salt levels across the 76 product categories for which salt reduction targets have already been set.

*Strengthening government leadership*: Public health advocates have argued that there is inadequate government leadership over the Deal—a fact illustrated by the exclusion of the FSA from its governance processes [75]. Weak government leadership permits food industry participants to exploit the conflict of interest between their profit objectives and the public health interest in achieving the national target for population salt intake [120,121,122]. Increasing the independence of the Deal’s administration and reducing industry influence over its governance structures is therefore a priority [56]. In Phase 1, this could be achieved by inviting other government agencies (including the FSA) to participate in high-level steering groups and committees, and by ensuring equal representation by government, industry and public health actors in the Deal’s governance structures. Changing the culture of the Deal’s governing bodies would signal to the food industry that if participants did not collectively achieve the targets for reformulation, more intrusive forms of regulation would follow.

*Increasing the integrity of the Deal’s performance monitoring process*: Currently, companies self-report on compliance with their pledges in an online form that documents the number of product categories that are meeting salt reduction targets, and the specific products within each category that meet current targets. In Phase 1, companies would be requested to report on the specific actions they had taken to incorporate the Deal’s requirements into their policies and procedures, and to identify the specific products that had been reformulated in accordance with their commitments. This data would flush out those companies that were “treading water,” enabling the Department of Health to audit the progress of poorly-performing companies, as well as participants’ progress overall.

*Increasing incentives to sign the salt reduction pledges*: The Deal must recruit more members in order to reduce overall levels of salt within the food supply, to prevent free-riders from deriving any business advantages that may arise from non-participation, and to maintain progress in adjusting consumers’ salt preference towards reduced-salt products [56,74]. In Phase 1, the Department of Health could promote the salt reduction pledges with greater urgency [77], and consider giving tax incentives to participants that join and make good faith attempts to reformulate their products. The Department could also undertake a targeted recruitment drive, identifying non-participating food manufacturers, retailers and caterers that contribute the greatest amount of salt to the food supply (based on sales data), and requesting that they join the deal. At the same time the Department could indicate that participation would become mandatory under Phase 2 if an insufficient number of companies joined the Deal.

*Disincentives for failing to participate*: Imposing penalties on participants for failing to comply with their commitments risks making it more difficult to recruit new participants to the Deal—and yet broad participation is needed if industry-driven salt reduction pledges are to deliver the progress that is required [13,77]. In Phase 1, the Department could sharpen enforcement efforts by “naming and shaming” companies that failed to meet the 2012 or 2017 targets within a given timeframe, while praising companies that met their targets through media releases. The threat of government action is an important incentive for action. The UK government has said that it will consider legislative alternatives to the Deal, but has not provided a detailed or time-bound statement of its intentions [75,77,123]. The Department of Health could announce that a failure to achieve 80% of the targets within three years would result in the introduction of maximum salt levels for a selection of high-volume products. Provided that it was committed to following through, this threat could strengthen the commitment of food businesses to the current version of the Deal, and increase momentum for reformulation efforts.

### 7.2. Phase 2

In Phase 2, large food manufacturers and retailers that exceeded a minimum turnover would be required to join the Deal (as well as smaller companies selling high-salt products), and maximum salt caps would be established for a greater range of product categories. While compliance with these caps could remain voluntary, products exceeding the caps would be required to carry a high-salt warning label. One way of implementing this might simply be to introduce mandatory traffic light labeling for processed foods and chain restaurant items. In addition, government might also consider imposing a tax on high-salt foods in categories where industry was failing to make adequate progress, using the revenues generated to fund consumer education campaigns.

Phase 2 would involve transferring administration of the Deal to an entity that was independent of industry, such as the FSA, thereby transforming the prevailing culture and reducing industry influence. This independent regulator would be responsible for setting salt reduction targets, (in consultation with the Deal’s networks and participating companies), and monitoring participants’ progress in meeting them. Although the average salt reduction targets for each category would also remain voluntary, the regulator could “name and shame” companies that made inadequate progress.

### 7.3. Phase 3

Phase 3 would trigger the imposition of a “two-track” regulatory approach, if an independent review showed that Phase 2 controls had failed to accelerate progress towards the national salt reduction target [124]. Participants making adequate progress in meeting targets would remain under the Deal’s salt reduction pledges. However, the independent regulator or the Department of Health would be authorized to publish specific targets for particular product categories or sub-categories that were mandatory for all participants. It could also require companies that continually failed to meet salt reduction targets to enter into enforceable undertakings with the Department to implement reformulation plans, accompanied by company-specific “catch-up” targets for specified products or product lines, and specifications for how these targets were to be achieved [75]. Non-compliant companies could be fined, and the publicity given to fines and the reputational damage to companies could be potent incentives towards compliance in most circumstances.

### 7.4. Phase 4

The selective use of regulatory scaffolds could be a powerful tool to change industry behavior, prompting companies to invest significant resources in meeting reformulation targets established under Phase 1 or 2. However, if the food industry’s level of engagement in reformulation continued to be unsatisfactory, the UK government could consider introducing a more comprehensive regulatory scheme. Sugarman has proposed performance-based regulation as one strategy, with significant fines imposed on supermarkets and retailers that failed to achieve targets for reductions in the overall volume of salt passing through their cash registers [113]. As shown in Figure 3, the government could also integrate supermarkets in reformulation efforts, imposing targets to encourage them to stock lower salt products and to pressure suppliers to reduce salt in leading brands. Alternatively, government could impose financial penalties on manufacturers that failed to meet mandatory sales-weighted, salt reduction targets, together with mandatory salt caps across a significant range of processed and restaurant food.

## 8. Conclusions

Voluntary efforts to achieve a national salt reduction target of 6 g/day have so far proved unsuccessful in the UK, while in Australia, the Food and Health Dialogue remains unlinked from any national target. In this paper we have presented a phased strategy for improving the performance of these voluntary programs, demonstrating how the theory of responsive regulation provides governments with a palate of options for strengthening under-performing, industry-led food reformulation efforts. The key features of this approach are summarized in Table 2. Necessary conditions for the success of our approach include setting national goals and targets, collecting baseline data on population salt intake in order to measure progress, and allocating responsibility for achieving a specified share of the national goal between processed food manufacturers and retailers, caterers and other participants in the voluntary scheme. Effective voluntary programs also require a genuine commitment from governments that they will increase the level of state supervision if food companies fail, collectively, to meet salt reduction targets within a given timeframe. Some of these conditions are present in the UK; none are currently present in Australia. Evidence suggests that significant mortality and disability might be avoided if governments were willing to strengthen voluntary food reformulation schemes with the organisational and regulatory “scaffolds” we have outlined in this paper.

A responsive strategy enables governments to draw upon market incentives for industry to improve the food environment voluntarily, while using the threat of further government action to encourage action by the food industry. Although governments can choose which regulatory controls are best suited to the goal of encouraging industry action, and need not impose all the controls discussed above, successive phases of interventions must, necessarily, impose stricter controls that industry has an incentive to avoid. Ultimately, performance matters. It is the relative lack of performance of current, voluntary processes that justifies the introduction of regulatory scaffolds, beginning with Phase 1, but proceeding towards a co-regulatory approach to salt reduction and ultimately towards mandatory standards and penalties, in a step-wise manner. Transparency is a significant feature of our approach, since transparency enables government to harness market pressures more effectively. Ultimately, the concept of legislative scaffolding provides a middle way for governments, enabling them to fulfill their public health obligations while avoiding (at least initially) the need for direct, statutory regulation of the food reformulation process.
